# Thiolated chitosan nanoparticles encapsulated nisin and selenium: antimicrobial/antibiofilm/anti-attachment/immunomodulatory multi-functional agent

**DOI:** 10.1186/s12866-024-03400-7

**Published:** 2024-07-12

**Authors:** Mozhgan Derakhshan-sefidi, Bita Bakhshi, Aliakbar Rasekhi

**Affiliations:** 1 Department of Bacteriology, Faculty of Medical Sciences, Tarbiat Modares University, Tehran, Iran; 2 Department of Biostatistics, Faculty of Medical Sciences, Tarbiat Modares University, Tehran, Iran

**Keywords:** Nanoparticles, Antimicrobial activity, Antibiofilm, Nisin, Selenium, Thiolated chitosan

## Abstract

**Background:**

The increase in the resistance of bacterial strains to antibiotics has led to research into the bactericidal potential of non-antibiotic compounds. This study aimed to evaluate in vitro antibacterial/ antibiofilm properties of nisin and selenium encapsulated in thiolated chitosan nanoparticles (N/Se@TCsNPs) against prevalent enteric pathogens including standard isolates of *Vibrio (V.) cholerae* O1 El Tor ATCC 14,035, *Campylobacter (C.) jejuni* ATCC 29,428, *Salmonella (S.) enterica* subsp. *enterica* ATCC 19,430, *Shigella (S.) dysenteriae* PTCC 1188, *Escherichia (E.) coli* O157:H7 ATCC 25,922, *Listeria (L.) monocytogenes* ATCC 19,115, and *Staphylococcus (S.) aureus* ATCC 29,733.

**Methods:**

The synthesis and comprehensive analysis of N/Se@TCsNPs have been completed. Antibacterial and antibiofilm capabilities of N/Se@TCsNPs were evaluated through broth microdilution and crystal violet assays. Furthermore, the study included examining the cytotoxic effects on Caco-2 cells and exploring the immunomodulatory effects of N/Se@TCsNPs. This included assessing the levels of both pro-inflammatory (IL-6 and TNFα) and anti-inflammatory (IL-10 and TGFβ) cytokines and determining the gene expression of *TLR2* and *TLR4*.

**Results:**

The N/Se@TCsNPs showed an average diameter of 136.26 ± 43.17 nm and a zeta potential of 0.27 ± 0.07 mV. FTIR spectroscopy validated the structural features of N/Se@TCsNPs. Scanning electron microscopy (SEM) images confirmed their spherical shape and uniform distribution. Thermogravimetric Analysis (TGA)/Differential Scanning Calorimetry (DSC) tests demonstrated the thermal stability of N/Se@TCsNPs, showing minimal weight loss of 0.03%±0.06 up to 80 °C. The prepared N/Se@TCsNPs showed a thiol content of 512.66 ± 7.33 µmol/g (*p* < 0.05), an encapsulation efficiency (EE) of 69.83%±0.04 (*p* ≤ 0.001), and a drug release rate of 74.32%±3.45 at pH = 7.2 (*p* ≤ 0.004). The synthesized nanostructure demonstrated potent antibacterial activity against various isolates, with effective concentrations ranging from 1.5 ± 0.08 to 25 ± 4.04 mg/mL. The ability of N/Se@TCsNPs to reduce bacterial adhesion and internalization in Caco-2 cells underscored their antibiofilm properties (*p* ≤ 0.0001). Immunological studies indicated that treatment with N/Se@TCsNPs led to decreased levels of inflammatory cytokines IL-6 (14.33 ± 2.33 pg/mL) and TNFα (25 ± 0.5 pg/mL) (*p* ≤ 0.0001), alongside increased levels of anti-inflammatory cytokines IL-10 (46.00 ± 0.57 pg/mL) and TGFβ (42.58 ± 2.10 pg/mL) in infected Caco-2 cells (*p* ≤ 0.0001). Moreover, N/Se@TCsNPs significantly reduced the expression of *TLR2* (0.22 ± 0.09) and *TLR4* (0.16 ± 0.05) (*p* < 0.0001).

**Conclusion:**

In conclusion, N/Se@TCsNPs exhibited significant antibacterial/antibiofilm/anti-attachment/immunomodulatory effectiveness against selected Gram-positive and Gram-negative enteric pathogens. However, additional ex-vivo and in-vivo investigations are needed to fully assess the performance of nanostructured N/Se@TCsNPs.

## Introduction

Prolonged exposure of bacteria to antimicrobial regimens can lead to acquired resistance, as all organisms have an instinctive tendency to adapt to their environment [1]. The development process of new antibiotics is facing many challenges due to their time-consuming and expensive nature [2]. In clinical settings, antibiotic resistance has been observed in both gram-positive and negative bacteria, with a greater prevalence seen among gram-negative strains due to the existence of an outer membrane permeability barrier that shields peptidoglycan and other intracellular targets from antimicrobial agents. This phenomenon has led to treatment complexities and the emergence of strains that are resistant to commonly used antibiotics [3].

Nisin is a polycyclic antibacterial peptide with broad-spectrum antibacterial activity. The compound demonstrated efficacy against a range of Gram-positive pathogens, although it also exhibited antibacterial activity against a limited number of Gram-negative pathogens [4]. The antimicrobial effectiveness of the lantibiotic nisin has been well established against bacterial pathogens, including methicillin-resistant *S. aureus* (MRSA), vancomycin-resistant enterococci (VRE), *L. monocytogenes*, *Bacillus cereus*, and *Clostridium botulinum* [5–7]. The interaction between nisin and the membrane-bound cell wall precursor lipid II and undecaprenyl pyrophosphate are shown to be the main antimicrobial mechanism of action [8]. This potent bacteriocin offers notable benefits, including its capacity to hinder bacterial growth at low concentrations, even in complex environments. Importantly, it has demonstrated a lower likelihood of promoting bacterial resistance in comparison to conventional antibiotics [9]. Gram-negative bacteria typically resist nisin, but instances of growth inhibition have been observed when the phospholipid structure within the bacterial cells is destabilized prior to exposure to nisin [6]. Many studies have investigated the increased antibacterial effect of nisin in combination with other compounds including poly-lactic acid against bacterial pathogens like *Micrococcus luteus* and *S. xylosus* [10]. Moreover, integration of nisin and 2,3-dihydroxybenzoic acid (DHBA) within nanofibers made from poly (D, L-lactide) (PDLLA) and poly (ethylene oxide) (PEO) has shown promising inhibitory effect on biofilm formation of methicillin-resistant *Staphylococcus aureus* (MRSA) [11]. Further investigations have highlighted the synergistic antibacterial and antibiofilm effects achieved when nisin is mixed with agents such as EDTA [12] and silver nanoparticles [13].

Selenium is considered a vital trace element with fundamental health benefits for both humans and animals. The antibacterial properties of selenium are based on its ability to induce oxidative stress in bacteria. This leads to the disruption of key cellular functions and potentially inhibits bacterial growth or causes cell death. These antimicrobial properties, combined with the immunomodulatory effects of this metalloid, make it a promising area of investigation for potential applications in the fight against bacterial infections [14]. Selenium nanoparticles (SeNPs) possess rapid absorption capabilities and minimal toxicity, rendering them safer options for use in conjunction with other treatments [15]. Research indicates that SeNPs can boost the antibacterial potency of standard antibiotics, resulting in a synergistic enhancement against a wide range of bacterial species, even those that are particularly hard to manage with antibiotics alone [16].

The most effective carrier vehicles for drug delivery among polymeric nanoparticles (NPs) are polysaccharides due to their exceptional physical and biological properties such as non-toxic, low immunogenicity and biocompatibility [17]. Chitin is the second most abundant naturally occurring polysaccharide after cellulose and can be converted to chitosan (Cs) through the process of N-deacetylation [18]. The mucoadhesive properties of modified Cs, in particular thiolated chitosan (TCs), in which thiol groups are immobilized on the primary amine group of Cs, that enables extended interaction between the drug and specific tissues, leading to improved drug absorption and availability within the body [19]. The bactericidal properties of Cs are due to its cationic nature, which enables it to attach to anionic elements on microbial surfaces. This feature makes it highly effective against pathogens [20]. *V. cholerae* O1 El Tor, *C. jejuni, S. enterica* subsp. *enterica, S. dysenteriae, E. coli* O157:H7, *L. monocytogenes*, and *S. aureus* are pathogenic bacteria known to cause gastroenteritis [21]. These bacteria have the ability to stimulate the immune system by interacting with *TLR2* and *TLR4* through their pathogen-associated molecular patterns. The activation of these receptors initiates a series of signaling events within the cells, ultimately triggering the innate immune response [22]. Inflammatory cytokines such as tumor necrosis factor-alpha (TNF-α), and interleukin-6 (IL-6) act as potent chemoattractant, effectively facilitating the recruitment and mobilization of immune cells towards the precise location of infection. This orchestrated interplay between inflammatory cytokines and immune cells substantiates their indispensable contribution in mounting an effective immune response against gastritis-causing bacterial infections [23]. However, excessive or prolonged overexpression of inflammatory cytokines can lead to tissue damage, systemic inflammation, and the development of inflammatory disorders. A finely balanced and controlled inflammatory response is crucial for pathogen clearance [24].

We propose that the combination of nisin with selenium will create a synergistic agent with both potent antibacterial and antibiofilm properties against common enteric pathogens such as *V. cholerae* O1 El Tor, *C.jejuni, S.enterica* subsp. *enterica, S.dysenteriae, E.coli* O157:H7, *L.monocytogenes*, and *S.aureus*. In this context, by using TC as a carrier and the synergistic effect of selenium, this study takes a pioneer approach to evaluate the controlled drug release effect of the obtained nanocomposite and to convey the limitations of degradation and absorption associated with free nisin in the gastroenteritis environment in the way of bactericidal application. We also anticipate that this evaluated nanocomposite will have anti-inflammatory attributes in addition to anti-bacterial effects. We aim to further explore the anti-biofilm potential of formulated nano-structures to inhibit bacterial attachment to the gut, potentially providing a novel strategy to address biofilm-associated infections.

## Experimental section

### Materials

Nisin (Molekula, UK), Chitosan (50–200 kDa) and Sodium tripolyphosphate (TPP, 85%) (Sigma‒Aldrich, USA), N-Hydroxysuccinimide (NHS) and N-Ethyl-N’-(3-dimethylaminopropyl) carbodiimide hydrochloride (EDAC. HCl) (Bio Basic Inc, Canada), ELISA kits (TNF-α, IL-6, IL-10, and TGF-β) (R& D, USA), MTT (3-(4,5-dimethylthiazol-2-yl)-2,5-diphenyltetrazolium bromide) assay kit (Yekta Tajhiz Azma, Iran), human colorectal adenocarcinoma cells (Caco-2) (Iranian Genetic Resources Center, Iran), Na_2_SeO_3_ and ascorbic acid (Merck, Germany) were purchased and utilized as directed. The highest-grade analytical reagents and other chemicals were used in this study.

### Strains of bacteria

Standard enteric pathogens were the focus of the current study. The strains used in this study were *V.cholerae* O1 El Tor ATCC 14,035, *C. jejuni* ATCC 29,428, *S.enterica* subsp. *enterica* ATCC 19,430, *S.dysenteriae* PTCC 1188, *E.coli* O157:H7 ATCC 25,922, *L.monocytogenes* ATCC 19,115, and *S.aureus* ATCC 29,733. Microbial strains were obtained from the microbiology bank of the Bacteriology Department, Faculty of Medicine of Tarbiat Modares University, Tehran, Iran, and all of them were confirmed by biochemical and molecular methods (16srRNA), and they were all kept at -80 °C in Brain Heart Infusion broth medium supplemented with 20% glycerol.

### Synthesize and characterization of N/Se@TCsNPs

**Fig. 1:**
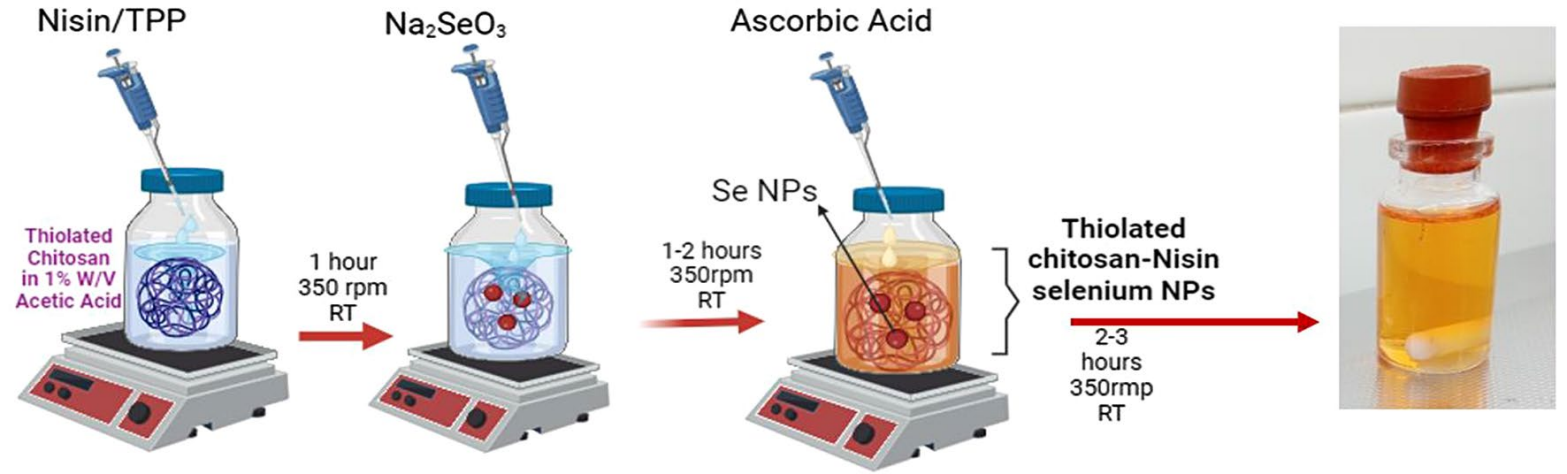
Schematic illustration of nanoparticle synthesis (Created with BioRender.com)

L-cysteine (l-Cys) (Sigma, USA) was functionalized and coupled to the Cs backbones using amide linkages and EDC/NHS as coupling agents [25]. Briefly, CsNPs (10 mg/mL) in 1% acetic acid (AA) solution was prepared for covalent thiol modification on Cs backbones. l-Cys (20 mg/mL) was functionalized with 1.5 mM of each EDC and NHS in distilled water, and then added to the CsNPs solution, with the resulting mixture agitated in the dark for 18 h (450 rpm, IKA^®^RH basic 2, Sigma, USA). To remove unbound residues, the prepared TCsNPs solution was dialyzed (cut-off 12 kDa MW, Sigma) prior to freeze-drying (Zirbus Vac 5, Germany). Nisin was encapsulated in TCsNPs by the ionotropic gelation method (Fig. 1). A solution of nisin (2.5 mg/mL) and TPP (1:10) was added dropwise to the TCsNPs solution (1% w/v in 1% AA solution) and the reaction mixture was stirred (450 rpm×1 h) [26]. The N/Se@TCsNPs were synthesized via the ionic reduction of Na_2_SeO_3_·5H_2_O using ascorbic acid as the reducing agent [27]. Na_2_SeO_3_ (5H_2_O) at a concentration of 1.2 mM was introduced dropwise to the N@TCsNPs solution. The resultant solution was then supplemented with ascorbic acid at a 4:1 ratio of ascorbic acid to Na_2_SeO_3_ (2 h×350 rpm, 25 °C). The final produced N/Se@TCsNPs were centrifuged (14,000 rpm×15 min, Sigma, Germany), freeze-dried (Zirbus Vac 5, Germany), and stored at 4 °C for further analysis.

To confirm the correct synthesis of the N/Se@TCsNPs, Fourier transform infrared spectroscopy (FTIR, PerkinElmer, USA; 400–4000 cm-1) was used at each step of the fabrication. The size distribution and zeta potential of the designed nanostructures were determined using a Zetasizer Nano ZS (Malvern, Worcestershire, UK) and their morphology was examined by scanning electron microscopy (SEM) using a VEGA3 TESCAN system at 20 kV. Thermogravimetric Analysis (TGA) and Differential Scanning Calorimetry (DSC) (TGA/DSC/1, METTLER TOLEDO. USA) techniques (5 °C/min from 25 °C to 200 °C) were also used to evaluate the thermal behavior of the formulated composites [28]. Iodometric titration was carried out to quantify the thiol groups incorporated in the N/Se@TCsNPs [29], using l-Cys- HCl solutions to construct the standard curve.

### In vitro entrapment efficacy (EE) and release behavior evaluation of N/Se@TCsNPs

An indirect method was used to determine the concentration of nisin entrapped in the N/Se@TCsNPs. Briefly, after the fabrication of the N/Se@TCsNPs, the supernatant obtained by centrifugation of the nanostructures (14,000 rpm, 15 min) was analyzed using the Pierce™ BCA Protein Assay Kit (Thermo Scientific, USA) as outlined in the manufacturer’s instructions [30].

The following formula is used to determine the amount of nisin present in formulated composites.

EE% = (Total nisin conc. in N/Se@TCsNPs – Supernatant nisin conc.)/ (Total nisin conc. in N/Se@TCsNPs) x100.

Inductively Coupled Plasma Atomic Absorption Spectroscopy (ICP-AAS) was also used to determine the EE% of selenium in the formulated NPs produced in accordance with the standard curve of Na_2_SeO_3_ [31].

The in vitro release behavior of nisin from the fabricated nanostructure was investigated over a period of 72 h. For this purpose, dialysis bags containing 1 mg of the synthesized N/Se@TCsNPs were prepared. The bags were then individually immersed in hydrochloric acid (pH = 3.2) and phosphate saline (PBS) (pH = 7.5). The aim of the study was to monitor the release of nisin from the N/Se@TCsNPs at specific time intervals, 1, 2, 3, 4, 24, 48 and 72 h, at 37 °C. Quantification of released nisin was performed using the Pierce™ BCA Protein Assay Kit [32].

### Stability evaluation of N/Se@TCsNPs

The stability of synthesised N/Se@TCsNPs was investigated to assess their response to temperature variation, contamination and degradation potential. To achieve this, the change in size and zeta potential was examined under controlled temperature conditions of 4 ± 2 °C and 25 ± 2.8 °C for a period of six months.

 [32]. In addition, to ensure the absence of contamination in the synthesized NPs, the sterility of both the synthesis conditions and the vessels used in the nanoparticle production process was verified. To this end, NPs were cultured on a number of media, including nutrient agar, blood agar, MacConkey agar and Sabouraud dextrose agar, and then placed under anaerobic and aerobic conditions to investigate the sterility of the NPs generated [33].

### In vitro cytotoxicity evaluation of N/Se@TCsNPs on Caco-2 cells

The cytotoxicity of the designed structure on Caco-2 cells was evaluated using the MTT colorimetric assay kit as directed by the manufacturer. Briefly, Caco-2 cells were seeded at a density of 5 × 10^4^ cells/well in a 96-well plate (SPL, Korea) with DMEM/F-12 medium supplemented with 10% FBS and Pen-Strep (10000 Units/mL) (Bioidea, Iran). The cells were incubated at 37 °C with 5% CO_2_ until they reached 85% confluence. The cells were subjected to treatment with N@TCsNPs, Se@TCsNPs and N/Se@TCsNPs at different concentrations (200-3.125 mg/mL), followed by a further incubation in a humidified incubator (37 °C, 5% CO_2_) for 24 h. MTT solution (0.5 mg/mL) was added to each well, followed by incubation at 37 °C for 3 h. The absorbance of formazan crystals dissolved in DMSO solvent (Sigma, USA) was then measured at 570–630 nm using an Epoch microplate reader (Biotek Instruments, USA). The 50% inhibitory concentration (IC_50_) was calculated based on triplicate experiments using positive control (PBS-treated cells in DMEM/F-12 medium) and negative control (DMEM/F-12 medium).

### In vitro study attachment and internalization study in presence of N/Se@TCsNPs

For the in vitro bacterial attachment study, 4 × 10^6^ Caco-2 cells/well were seeded on 96-well plates until they reached 80% confluence. Formulated N/Se@TCsNPs at IC_50_ concentration were then added to the wells. A 100 µL suspension of each isolate was prepared in sterile PBS (pH = 7.2) at their MOI of 7, 50, 100, 50, 100, 100 and 50 CFU/100 µL for *V. cholerae* O1 El Tor, *C. jejuni*, *S. enterica subsp. enterica*, *S. dysenteriae*, *E. coli* O157:H7, *L. monocytogenes* and *S. aureus*, respectively, and the plates were incubated for 3 h in a humidified incubator (37 °C, 5% CO_2_). To assess the number of bacteria adhering to Caco-2 cells, cells were washed three times with PBS after time, lysed with 0.01% Triton-X100 (Sigma, USA) and serially diluted on Brain Heart Infusion (BHI) agar (Sigma, USA). A separate set of confluent wells was used to further investigate bacterial internalization into Caco-2 cells in the presence of N/Se@TCsNPs. The procedure was the same as described above, except that to remove adherent bacteria, 100 µg/mL gentamycin was used. The cells were then lysed with 0.01% TritonX-100 and cultured on BHI agar in serial dilutions to determine the number of invading bacteria. All experiments were performed in three independent experiments. Positive control (bacteria + Caco-2 cells in DMEM/F-12 medium) and negative control (PBS-treated cells in DMEM/F-12 medium) were included [34].

### In vitro *TLR2* and *TLR4* gene expression evaluation in Caco-2 cells

The effect of N/Se@TCsNPs on *TLR2* and *TLR4* gene expression in Caco-2 cells in the presence of *V. cholerae* O1 El Tor, *C*. *jejuni, S. enterica subsp. enterica, S. dysenteriae, E. coli* O157:H7, *L. monocytogenes* and *S. aureus* was evaluated by quantitative real-time PCR (qRT-PCR). For this purpose, 5 × 10^6^ Caco-2 cells/well were cultured in 24-well polystyrene microplates (SPL; Korea) and incubated (37 ºC, 5% CO_2_) until reach 85% confluence. The cells were then exposed to the MOI CFU of each bacterium for 1 h. After this incubation period, the Caco-2 cells extracellular bacteria and medium were carefully replaced with DMEM/F-12-free compounds, including the addition of N/Se@TCsNPs (at IC_50_ concentration). After the incubation period (18 h, 37ºC, 5% CO_2_) the supernatant (liquid part) was removed and following three times washing with PBS, the Caco-2 cells were used for RNA extraction and complementary DNA (cDNA) synthesis using the Total RNA mini kit and cDNA synthesis kit (Yekta Tajhiz Azma, Iran), respectively, as per the manufacturer’s instructions. The QRT-PCR amplification process for TLR2 and TLR4 genes was carried out utilizing an Applied Biosystems Step One Plus real-time fluorescence thermal cycler. This procedure involved conducting PCR amplification on each bacterial isolate under two conditions: initially, as a control, without exposure to N/Se@TCsNPs; and subsequently, following exposure to fabricated nanostructure (Table 1). The results were analyzed with the 2^−ΔΔCT^ method in terms of *GAPDH* expression levels [35].

**Table 1:** Primers used in this study

Primer Targets	Sequences (5’-3’)	Amplification conditions	Amplification product size (bp)	Ref.
		94 °C (1 min)		
*TLR2*	F: ATCCTCCAATCAGGCTTCTCT R: ACACCTCTGTAGGTCACTGTTG	53 °C (30 s) 72 °C (1 min)	163	[36]
		94 °C (1 min)		
*TLR4*	F: AAGCCGAAAGGTGATTGTTG R: CTGAGCAGGGTCTTCTCCAC	53 °C (30 s) 72 °C (1 min)	143	[37]
		94 °C (1 min)		
*GAPDH*	F: GAGCCACATCGCTCAGACAC R: CATGTAGTTGAGGTCAATGAAGG	58 °C (30 s) 72 °C (1 min)	108	[36]

### Inflammatory (TNF-α and IL-6) and anti-inflammatory (TGF-β and IL-10) cytokines evaluation

The supernatant of the Caco-2 cells was collected at 6 and 24 h after treatment with the designed NPs and stored at -80 °C to investigate the impact of N/Se@TCsNPs on the production of inflammatory (TNF-α and IL-6) and anti-inflammatory (TGF-β and IL-10) cytokines by using sandwich ELISA approach by ELISA kit (R&D, USA) during the cell culture stage. The amount of cytokines was determined using standard curves created with fixed cytokine concentrations. A microplate spectrophotometer was used to determine the optical density (OD_450_ nm).

### Antibacterial/antibiofilm activity of N/Se@TCsNPs evaluation using the microbroth dilution/ crystal violet assays

The minimum inhibitory concentration (MIC) of N/Se@TCsNPs was determined following the guidelines established by the Clinical and Laboratory Standards Institute (CLSI) [38]. The N@TCsNPs, Se@TCsNPs, and N/Se@TCsNPs were 2-fold serially diluted in Muller Hinton broth (MHB) in a 96-well microtiter plate, with the final concentrations ranging from 200 to 1.5 mg/mL. A final each bacterial concentration equivalent to 0.5 McFarland standard solutions (OD_620_ nm) was then introduced into the wells. Broth medium containing the same number of bacteria served as positive control, while blank broth medium used as negative control. The plates were sealed and incubated for 18–24 h in a shaking incubator at 37 °C (IKA@KS, Sigma, USA). The MIC was calculated by measuring the optical density of bacterial growth in wells treated with nanostructures compared to the control wells (OD_620_ nm). In addition, 100 µL of the supernatant from each well was cultured on a BHI agar medium to count the number of viable bacteria exposed to the synthesized NPs. To calculate the MIC of the synthesized nanoparticle for each bacterium, our initial step involved measuring the optical density (OD) of bacterial samples exposed to different nanoparticle concentrations at a wavelength of 620 nm. These readings were then subtracted from the OD of the blank well. Additionally, we compared these values with the optical densities of the positive control wells and also considered the colony counts from each dilution plated on BHI agar medium. Based on this comprehensive analysis, the MIC was calculated.

The biofilm inhibitory effect of newly fabricated N/Se@TCsNPs was studied according to the microdilution crystal violet assay in a 96-well polystyrene microplate. Briefly, the wells of 96-well plates were inoculated with 100 µl of broth media, along with 0.5% (w/v) sucrose, 10 µl of bacterial suspension (10^8^ CFU/mL), and 100 µl of different 2-fold concentrations of N@TCsNPs, Se@TCsNPs, and N/Se@TCsNPs. After the desired incubation period (37 ºC, 24 h), by staining the wells with 1% (wt/vol) crystal violet solution, the biofilm inhibition rate was measured (OD _570_ nm). To compare the impact of fabricated N/Se@TCsNPs on the biofilm growth of various bacteria, positive control (wells with the same bacterial count but without NPs) and negative control (empty wells filled only with broth medium) were employed. The OD (570 nm) measurements revealed that greater densities corresponded to thicker biofilms, signifying a larger number of bacterial cells adhering to the surface at the time of crystal violet staining. On the other hand, lower optical densities pointed towards fewer bacterial cells, suggesting either a reduction in biofilm thickness or diminished biofilm formation.

To determine planktonic cells of *V.cholerae* O1 El Tor, *S.enterica* subsp. *enterica*, *S.dysenteriae*, *E.coli* O157:H7, *L.monocytogenes*, *S.aureus*, and *C.jejuni* after specific time intervals of biofilm formation and exposure to constructions, the cell suspension were analyzed compare to the OD_595_ nm biofilm inhibition positive and negative control wells. Each test was carried out in triplicate.

### Statistical analysis

Each experiment was conducted in triplicate, with the outcomes reported as the mean ± standard deviations. Experimental and control groups were compared by one-way analysis of variance (ANOVA), followed by Tukey’s post hoc and Sidak’s multiple comparisons test. Calculations were performed using the GraphPad Prism Software version 9.5 (GraphPad Software, Inc, La Jolla, CA, USA) to determine the differences that were statistically significant (*p*-value˂0.05).

## Results

### Characterization of the designed N/Se@TCsNPs

**Fig. 2:**
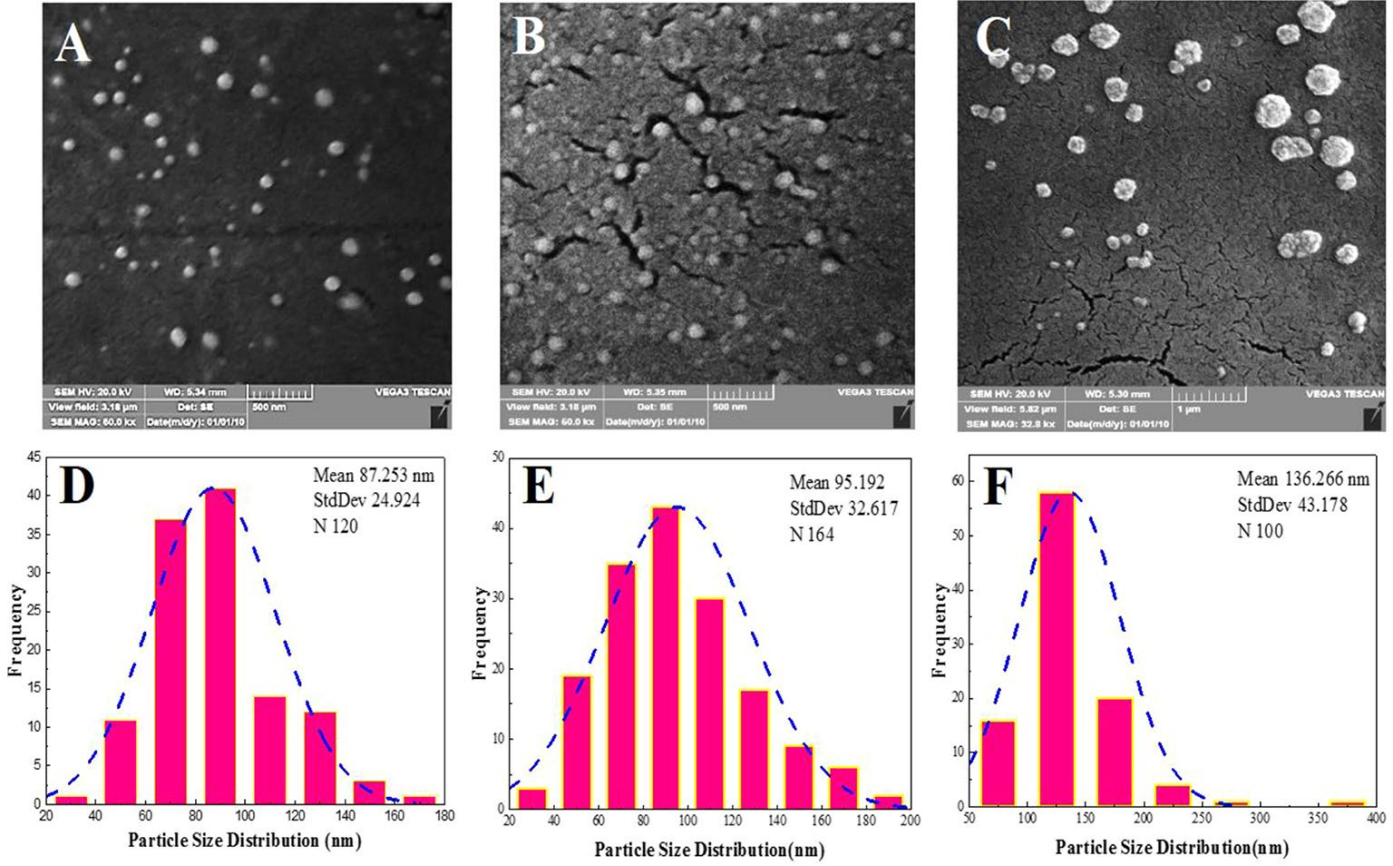
Morphology and size distribution of N/Se@TCsNPs: scanning electron microscopy imaging of fabricated nanostructures, **A** N@TCsNPs, **B** Se@TCsNPs, **C** N/Se@TCsNPs, and histogram of particle size distribution **D** N@TCsNPs, **E** Se@TCsNPs, and **F** N/Se@TCsNPs. **N@TCsNPs**: Nisin encapsulated in thiolated chitosan nanoparticles, **Se@TCsNPs**: Selenium encapsulated in thiolated chitosan nanoparticles, and **N/Se@TCsNPs**: Nisin and selenium encapsulated in chitosan nanoparticles

**Fig. 3:**
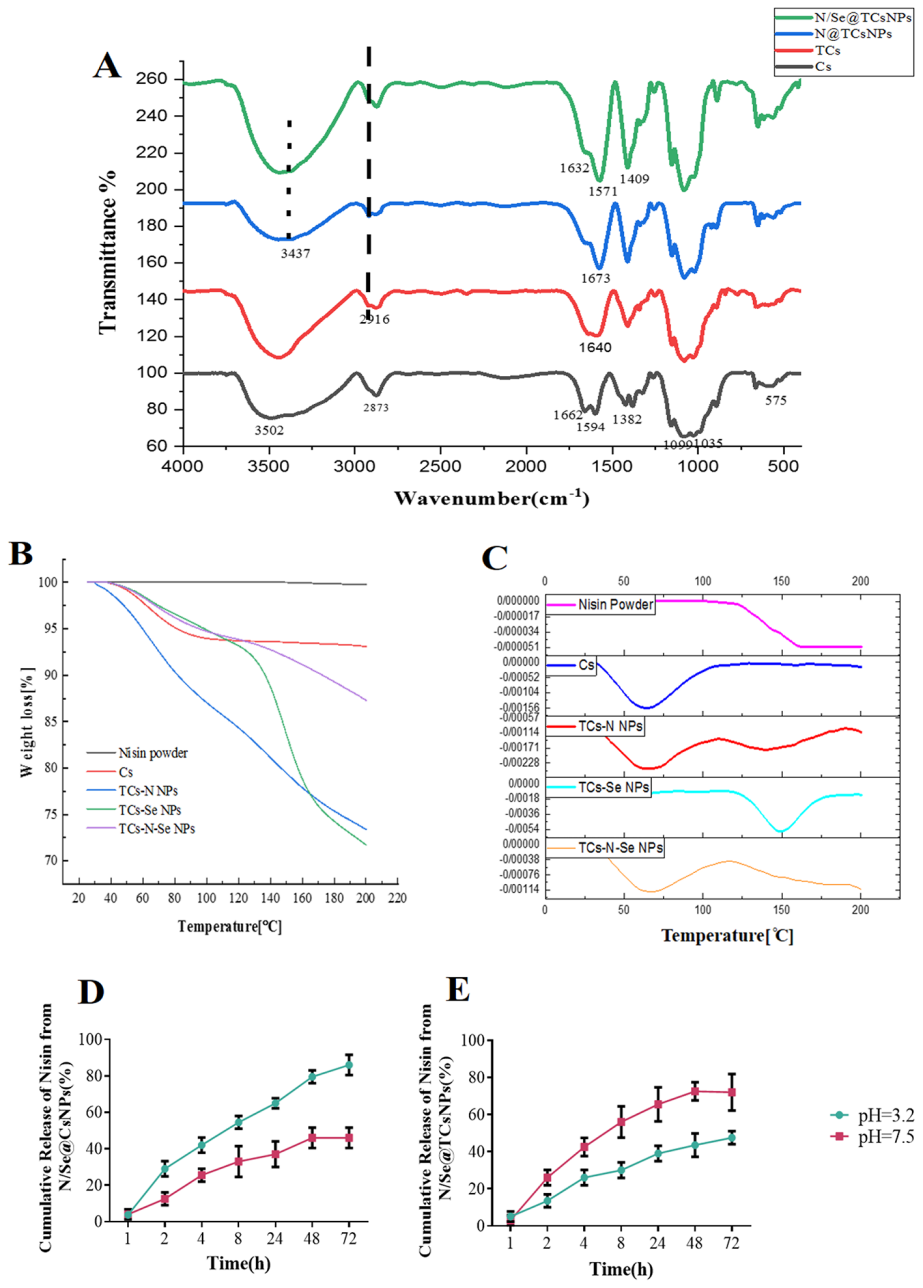
Physicochemical characterization of fabricated nanostructures. **A** Fourier Transform Infrared (FTIR) spectra of Cs, TCs, N@TCsNPs, and N/Se@TCsNPs. **B** Differential Scanning Calorimetry diagram of weight changes in the N/Se@TCsNPs. **C** Thermogravimetry diagrams; derivatives of weight changes in the N/Se@TCsNPs components, and the release profile of nisin from **D** N/Se@CsNPs, and **E** N/Se@TCsNPs at various pH levels. **Cs**: Chitosan, **TCs**: Thiolated chitosan, **N@TCsNPs**: Nisin encapsulated in thiolated chitosan nanoparticles, **Se@TCsNPs**: Selenium encapsulated in thiolated chitosan nanoparticles, and **N/Se@TCsNPs**: Nisin and selenium encapsulated in chitosan nanoparticles

The N/Se@TCsNPs exhibited a size of 136.26 ± 43.178 nm and positive surface charge of 0.271 ± 0.07 mV (Table 2). SEM imaging also revealed spherical shape of N/Se@TCsNPs with smooth surfaces (Fig. 2). The FTIR spectra of Cs at different manufacturing phases reveal distinct absorption peaks linked to carbonyl and amine groups. The amide groups are linked to asymmetric and symmetric stretching vibrations at 1660 and 1382 cm^− 1^. The saccharide structure of Cs is connected to absorption peaks in the 1000–1200 cm^− 1^ range. The Cs ring experiences bending vibrations at approximately 575 cm^− 1^. The amide carbonyl group band is at 1652 cm^− 1^, while the pyranose ring’s C-H and C-O stretching are represented by bands at 2873 and 1035 cm^− 1^. These thiolate groups can also introduce additional C-S-H stretches in the spectrum, which can be detected in the 2700–2500 cm^− 1^ region. Moreover, the presence of nisin and its integration into the N/Se@TCsNPs through potential physicochemical interactions account for the higher intensity of N-H and O-H stretching vibrations that was recorded at approximately 3437 cm^− 1^. Nisin-incorporated TCsNPs exhibited greater intensity in the absorption band at 1673 cm^− 1^ due to C = O stretching vibration and a slight shift of approximately 33 cm^− 1^. The transfer of absorption peaks associated with carbonyl groups from 1662 to 1382 cm^− 1^ and those associated with amine groups from 1594 cm^− 1^ in pure Cs to 1632, 1409, and 1571 cm^− 1^ in the N/Se@TCsNPs sample, respectively, is shown in the presence of selenium in N/Se@TCsNPs (Fig. 3A).

**Table 2:** Physiochemical characterization of optimized N/Se@TCsNPs, N/Se@TCsNPs: nisin and selenium encapsulated in chitosan nanoparticles; **cs**: Chitosan, **TCs**: Thiolated Chitosan, **N@TCsNPs**: Nisin encapsulated in thiolated chitosan nanoparticles, and **N/Se@CsNPs**: Nisin and selenium encapsulated in chitosan nanoparticles

Formulation	Cs (mg/mL)	Cysteine (mg/mL)	Nisin (mg/mL)	Selenium (µg/mL)	PH	Z-average size (nm)	PDI	Thiol content µmol/g
TCs І	10	10	-	-	5	-	-	484.3 ± 9.01
TCs ІІ	10	15	-	-	5	-	-	495 ± 5.0
TCs ІІІ	10	20	-	-	5	-	-	512.66 ± 7.33
N@TCsNPs І	10	20	2	-	7	59.6 ± 0.52	0.49 ± 0.05	512.66 ± 7.33
N@TCsNPsІІ	10	20	2.5	-	7	87.25 ± 24.92	0.25 ± 0.03	512.66 ± 7.33
N@TCsNPsІІІ	10	20	5	-	7	precipitation	-	-
N/Se@TCsNPsІ	10	20	2.5	200	7	136.26 ± 43.17	0.27 ± 0.07	512.66 ± 7.33
N/Se@TCsNPsІІ	10	20	2.5	250	7	precipitation	-	-
N/Se@TCsNPsІІІ	10	20	205	300	7	Aggregation immediately	-	-

Figure 3B and C has shown the graph of weight loss variations as a result of temperature increases in both N/Se@CsNPs and N/Se@TCsNPs. As shown in Fig. 3B the evaporation of small molecules in N@TCsNPs and Cs samples occurred at approximately 65 °C. However, for Se@TCsNPs, the evaporation of small molecules took place at 150 °C. A single significant thermal degradation peak at 165 °C was observed in the TGA study of pure nisin. The final TGA curve of N/Se@TCsNPs had two peaks; the first peak began at 65 °C and persisted until 80 °C, equating to a weight loss of 0.03%, which can be attributed to moisture loss. The second shift in the curve began at 198̊C and is attributed to the significant weight loss caused by the dissolution of C-H bonds, which refers to the breakdown and deterioration of the nanocomposite. The free thiol content and of N/Se@TCsNPs was 89.12 ± 4.23 µM/g (1 mM iodine, indicator: starch y = 0007x + 0.2268/R^2^=0.97.7).

### In vitro entrapment efficacy (EE) and release behavior evaluation of N/Se@TCsNPs

The study found that the EE% of nisin in N/Se@TCsNPs was 69.83%±0.04 at pH = 7.2. Using the ICP method, the selenium concentration in the nanostructure was determined to be 0.65 ± 0.01 µg/mL (y = 100.55x-62.5, R^2^ = 0/99).

The release profile of nisin from formulated nanostructures was observed to occur in two phases. At alkaline conditions (pH = 7.5), an initial rapid release was observed, with nisin being released from N/Se@TCsNPs at a significantly faster rate compared to N/Se@CsNPs (Fig. 3D and E). Nisin release from N/Se@TCsNPs exhibited an initial burst release phase within the first 10 h, during which approximately 51.39% ± 10.96 of the nisin was released. Following this rapid release, a prolonged, sustained release occurred over the subsequent 72 h, accounting for about 80.53% ± 6.109 of the total nisin release. Meanwhile, the release pattern of N/Se@CsNPs adhered to a non-Fickian mechanism, as supported by the calculated (R^2^) coefficient (0.77 ± 0.01), signifying that the release was influenced by both diffusion and relaxation processes throughout the 72 h period.

### Stability evaluation of N/Se@TCsNPs

During an extensive 6-month study, the particle sizes at temperatures of 4 ± 2 °C and 25 ± 3 °C were 122 ± 0.03 nm and 139 ± 0.73 nm, respectively. The zeta potential values of the N/Se@TCsNPs at temperatures of 4 ± 2ºC and 25 ± 3ºC were 1.30 ± 0.08 and 7.06 ± 0.04 mV, respectively. At 4 ± 2 °C, compared to higher temperatures (25 ± 3 °C), the resulting composite was more stable. In this experiment, N@TCsNPs, Se@TCsNPs, and N/Se@TCsNPs were cultivated. No bacterial growth was observed under anaerobic and aerobic conditions on thioglycolate media, nutritional agar, blood agar, Macconkey agar, and Sabouraud dextrose agar media after 24 and 48 h, indicating that the produced samples were sterile.

### In vitro cytotoxicity evaluation of N/Se@TCsNPs on Caco-2 cells

**Fig. 4:**
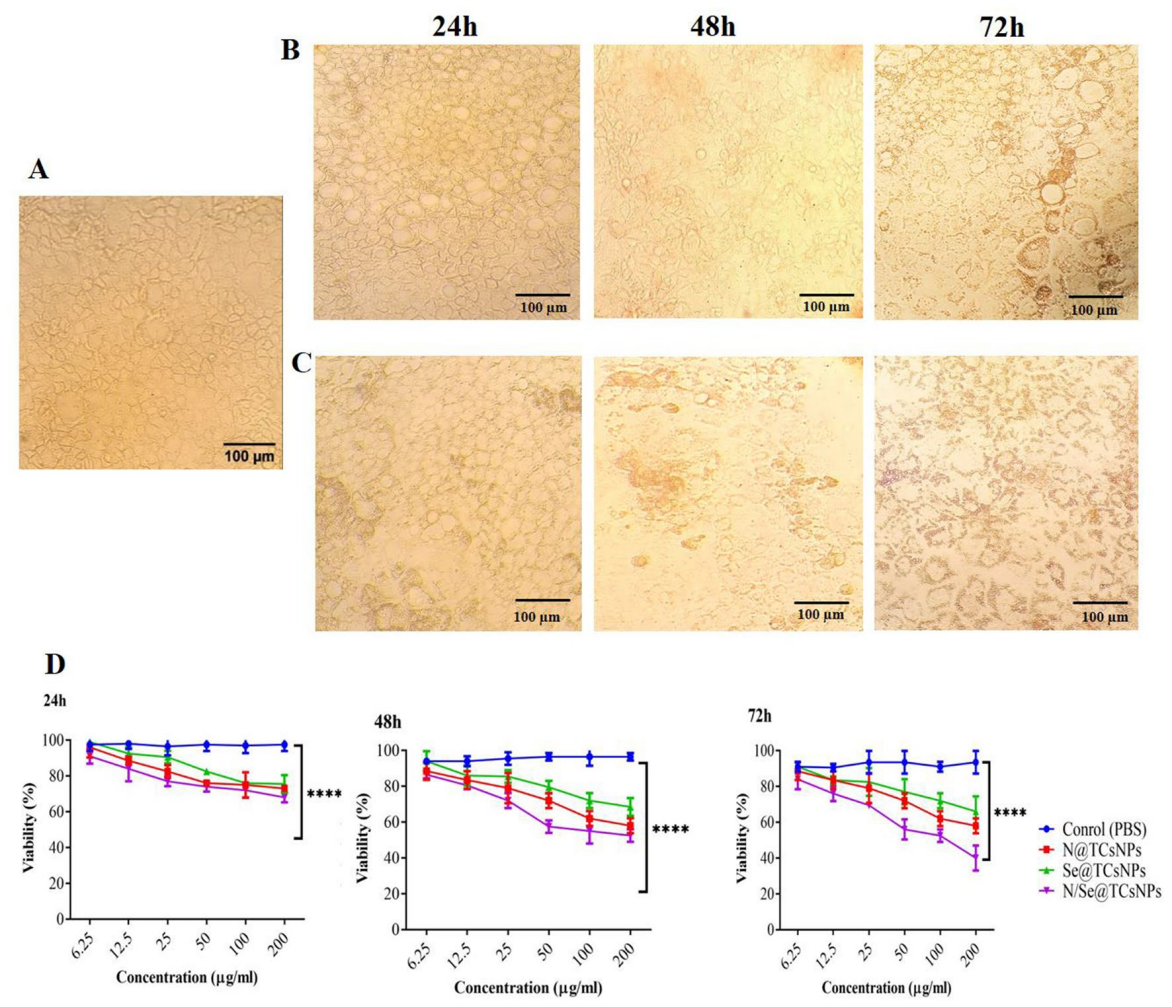
Caco-2 cells treated with N/Se@TCsNPs at intervals of 24, 48, and 72 h under an inverted microscope (20x). **A** 25 mg/mL and **B** 200 mg/mL. **C** Cell viability in the MTT test following treatment with formulated NPs. **N@TCsNPs**: Nisin encapsulated in thiolated chitosan nanoparticles. **Se@TCsNPs**: Selenium encapsulated in thiolated chitosan nanoparticles, and **N/Se@TCsNPs**: Nisin and selenium encapsulated in chitosan nanoparticles, **MTT**: (3-(4,5-dimethylthiazol-2-yl)-2,5-diphenyltetrazolium bromide

Exposure of Caco-2 cells to varying concentrations of N@TCsNPs, Se@TCsNPs, and N/Se@TCsNPs resulted in evident alterations in cell morphology. In particular, treating cells with a concentration of 200 mg/mL of N/Se@TCsNPs resulted in necrosis after 24 h, evidenced by cell lysis and the discharge of cellular components into the surroundings. This process can result in the deterioration of cell integrity and morphology. By 72 h following the treatment, the cell walls entirely disintegrated, and its contents spilled out. On the other hand, at a concentration of 25 mg/mL, the smallest alterations in cell morphology were noted within 48 h (Fig. 4A-C). The IC_50_ values for N/Se@TCsNPs were found to be 447.46 ± 16.57 mg/mL, 80.04 ± 10.94 mg/mL, and 69.60 ± 4.68 mg/mL at 24, 48, and 72 h, respectively. These findings underscore the synergistic impact of nisin and selenium in the homogeneous composite, demonstrating superior efficacy compared to their individual effects (Fig. 4D).

### In vitro attachment and internalization study in presence of N/Se@TCsNPs

**Fig. 5:**
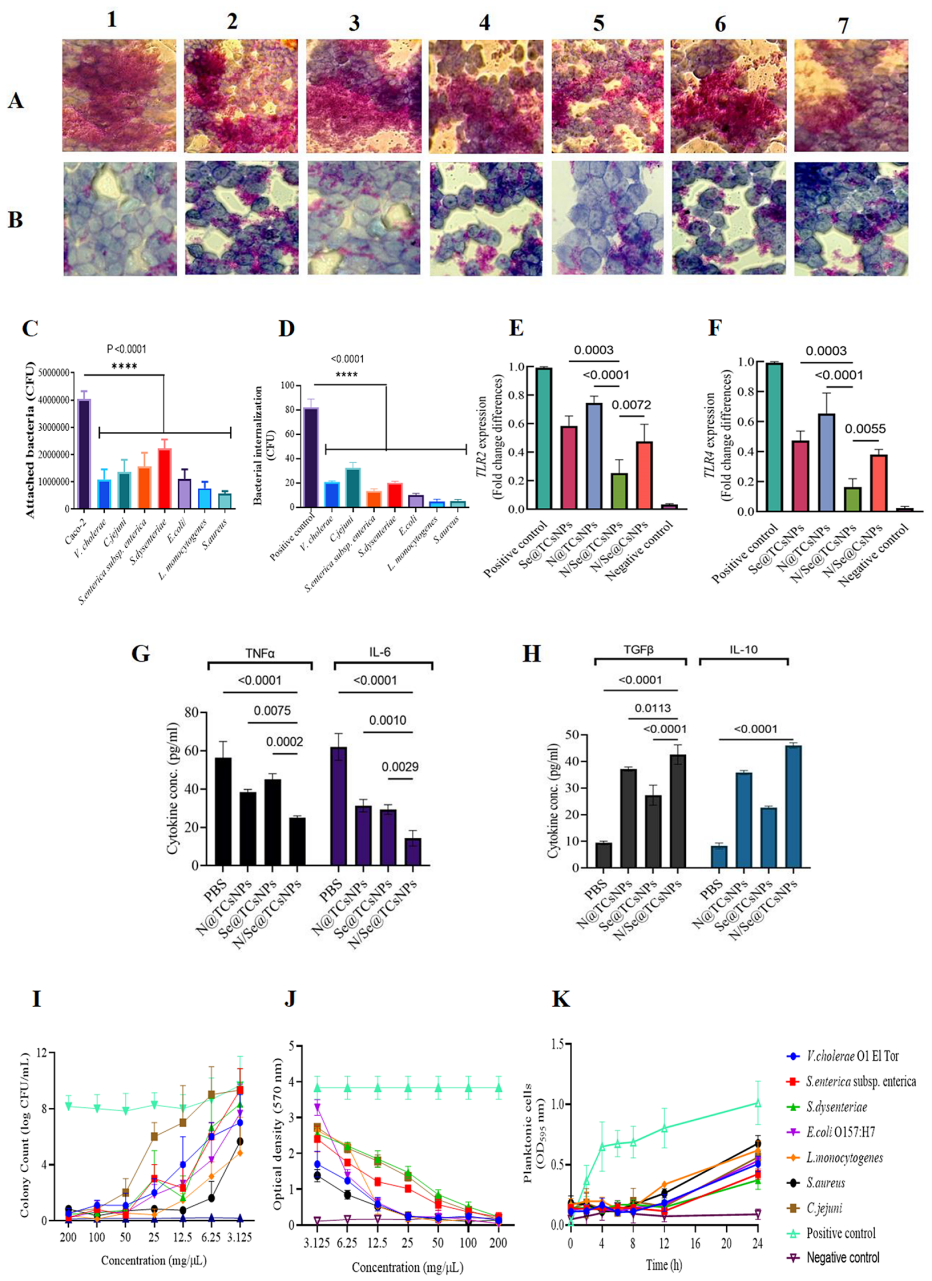
Attachment study of enteric pathogens to Caco-2 cells under light microscopy with Giemsa stain, ×100. **A** positive control. **B** Treatment with N/Se@TCsNPs; (1) *V. cholerae* O1 *El Tor*, (2) *C.jejuni*, (3) *S.enterica* subsp. *enterica*, (4) *S.dysenteriae*, (5) *E.coli* O157:H7, (6) *L.monocytogenes*, and (7) *S.aureus.***C** Attached bacteria rates represented wrt control. **D** Internalization rate of bacteria; gene expression evolution in response to N/Se@TCsNPs by quantitative real-time PCR technique. **E***TLR2* and **F***TLR4*, positive control (Caco-2 + bacteria) and negative control (Caco-2 cells + PBS); immunomodulatory effects of N/Se@TCsNPs. **G** IL-6, and TNF-α after 6 h **H** IL-10, and TGF-β after 24 h treatment. **I** Colony count of bacteria to evaluate the minimum inhibitory concentration of nano-construction. **J** Biofilm inhibition evaluation by UV assay (OD_595_nm). **K** alterations noted in the optical density of planktonic cells in response to nano-treatments. The data points shown represent the average outcomes from three separate experiments (m ± SD) (*p* < 0.05). **N@TCsNPs**: Nisin encapsulated in thiolated chitosan nanoparticles, **Se@TCsNPs**: Selenium encapsulated in thiolated chitosan nanoparticles, and **N/Se@TCsNPs**: Nisin and selenium encapsulated in chitosan nanoparticles, **Caco-2**: Human colorectal adenocarcinoma cells

The results of the attachment analysis (Fig. 5A-C) demonstrated a significant reduction in the number of attached bacteria when N/Se@TCsNPs were present in the Caco-2 cell culture medium. In the absence of N/Se@TCsNPs, the number of bacteria attached was 4,050,000 ± 278,388 CFU/mL. However, with the inclusion of N/Se@TCsNPs, the numbers decreased to 1,093,000 ± 368,963 CFU/mL for *V. cholerae* O1 El Tor, 1,357,000 ± 455,668 CFU/mL for *C. jejuni*, 1,567,000 ± 503,322 CFU/mL for *S.enterica* subsp. *enterica*, 2,233,000 ± 321,455 CFU/mL for *S.dysenteriae*, 1,107,000 ± 352,326 CFU/mL for *E.coli* O157:H7, 766,667 ± 241,316 CFU/mL for *L.monocytogenes*, and 573,333 ± 87,369 CFU/mL for *S.aureus* (p˂0.0001).

Invaded bacterial in internalization results (Fig. 5D) revealed antibiofilm and antibacterial effect of N/Se@TCsNPs. The bacterial internalization in exposure of obtained nanocomposite were 19 ± 1.00, 31.67 ± 2.93, 12.67 ± 1.45, 22 ± 1.52, 9.33 ± 1.20, 4 ± 1.52, and 4.25 ± 1.10 CFU/mL for *V. cholerae* O1 El Tor, *C. jejuni*, *S.enterica* subsp. *enterica*, *S.dysenteriae*, *E.coli* O157:H7, *L.monocytogenes*, and *S.aureus*, respectively (p˂0.0001).

### In vitro *TLR2* and *TLR4* gene expression evaluation in Caco-2 cells

In the presence of both selected gram-negative and positive isolates, the exposure of Caco-2 cells to N/Se@TCsNPs led to a significant reduction in the expression of *TLR2* and *TLR4* genes. The *TLR2* gene expression showed a decrease of 74.60%±0.04, 58.50%±0.06, 25.37%±0.09, and 47.53%±0.12 when treated with N@TCsNPs, Se@TCsNPs, N/Se@TCsNPs, and N/Se@CsNPs, respectively. Similarly, the *TLR4* gene expression was reduced by 65.50%±0.13, 47.33%±0.06, 16.33%±0.05, and 38.00%±0.03 with the respective treatments mentioned above (*p*˂ 0.05). Notably, N/Se@TCsNPs exhibited a more pronounced inhibitory effect on reducing *TLR2* and *TLR4* gene expression compared to all other components (Fig. 5E and F).

### Inflammatory (TNF-α and IL-6) and anti-inflammatory (TGF-β and IL-10) cytokines evaluation

The introduction of N/Se@TCsNPs significantly reduced the concentrations of pro-inflammatory cytokines IL-6 (to 14.33 ± 2.33 pg/mL) and TNFα (to 25 ± 0.5 pg/mL) in Caco-2 cells that were infected with bacteria (*p* < 0.0001). Contrarily, this treatment caused a considerable increase in the production of anti-inflammatory cytokines IL-10 (to 46.00 ± 0.57 pg/mL) and TGFβ (to 42.58 ± 2.10 pg/mL) in the same infected Caco-2 cells (*p* < 0.0001) (Fig. 5G and H).

### Antibacterial/ antibiofilm activity of N/Se@TCsNPs evaluation using the microbroth dilution/ crystal violet assays

Our findings indicate that pure nisin and N@TCsNPs demonstrated reduced effectiveness against Gram-negative bacteria compared to Gram-positive bacteria. Nevertheless, N/Se@TCsNPs exhibited improved antibacterial capabilities, particularly with enhanced drug release, showing efficacy in the range of 1.5 ± 0.08-25 ± 4.04 mg/mL across both types of bacteria. The colony counts of viable bacteria treated with N/Se@TCsNPs further highlighted its antibacterial properties at concentrations above 3.125 mg/mL (6.25 ± 0.53-25 ± 4.04 mg/mL), significantly inhibiting the growth of *V. cholerae* O1 El Tor, *C. jejuni*, *S. enterica subsp. enterica*, *S. dysenteriae*, and *E. coli* O157:H7 (Fig. 5I). Additionally, the antibacterial action of N/Se@TCsNPs intensified at higher concentrations, although it effectively suppressed the growth of *L. monocytogenes* and *S. aureus* at lower concentrations of 3.125 ± 0.19 and 1.5 ± 0.08 mg/mL, respectively. Interestingly, our research did not detect any significant antimicrobial benefits of TCs over Cs-coating against bacterial strains (Table 3).

**Table 3:** Minimum inhibitory concentration values (mg/mL) of Se@TCsNPs, N@TCsNPs, N/Se@TCsNPs, N/Se@CsNPs, pure nisin and sodium selenite against microbial strains, **N/Se@TCsNPs**: Nisin and selenium encapsulated in thiolated chitosan nanoparticles, **N/Se@CsNPs**: Nisin and selenium encapsulated in chitosan nanoparticles, **Se@TCsNPs**: selenium encapsulated in thiolated chitosan nanoparticles

Microorganism type	Strain	Se@TCsNPs	*N*@TCsNPs	*N*/Se@TCsNPs	*N*/Se@CsNPs	Nisin	Sodium selenite
gram-positive	*S aureus*	25 ± 0.05	12.5 ± 0.32	1.5 ± 0.08	1.5 ± 0.4	25 ± 0.01	100 ± 0.04
	*L. monocytogenes*	50 ± 0.16	12.5 ± 0.48	3.125 ± 0.19	6.25 ± 0.02	25 ± 0.07	200 ± 0.05
gram-negative	*E. coli*	25 ± 0.42	50 ± 0.75	6.25 ± 0.53	12.5 ± 0.01	200 ± 0.02	˃500
	*S. enterica.* Sub. *enterica*	100 ± 0.04	200 ± 0.01	12.5 ± 0.26	12.5 ± 5.03	˃500	˃500
	*S.dysenteriae*	50 ± 0.05	100 ± 0.35	6.25 ± 0.05	50 ± 4.61	˃500	˃500
	*V.cholerae* O1 El Tor	25 ± 0.01	100 ± 4.29	12.5 ± 1.01	25 ± 0.04	˃500	˃500
	*C. jejuni*	50 ± 0.01	200 ± 10.38	25 ± 4.04	25 ± 0.07	˃500	˃500

The study revealed that N/Se@TCsNPs possess inhibitory effects on the biofilm formation of S. aureus and *L. monocytogenes* at concentrations exceeding 6.25 mg/mL. Notably, at a concentration greater than 25 mg/mL, it effectively prevented biofilm formation in *V. cholerae* O1 El Tor, *C. jejuni*, *S. enterica subsp. enterica*, *S. dysenteriae*, and *E. coli* O157:H7 (Fig. 5H). UV assay statistical analysis indicated that N/Se@TCsNPs nearly eliminated biofilm formation by 100% for both *S. aureus* and *L. monocytogenes*. Moreover, the nanocomposite at 25 ± 0.17 mg/mL significantly reduced biofilm formation by about 60% against the aforementioned gram negative pathogens (*V. cholerae* O1 El Tor, *C. jejuni*, *S. enterica subsp. enterica*, *S. dysenteriae*, and *E. coli O157:H7*) (Fig. 5J). The antibiofilm activity of N/Se@TCsNPs was notably stronger at higher concentrations (200, 100, and 50 mg/mL) compared to lower ones. Biofilm formation was categorized as non-adherent at 200 and 100 mg/mL, weak at 50 and 25 mg/mL, and strong at 12.5, 6.25, 3.125, and 0 mg/mL, with no intermediate levels observed among the selected bacteria. Importantly, nisin or selenium alone in TCsNPs were effective against biofilm formation at significantly higher concentrations than when administered together as uniform N/Se@TCsNPs (*p* < 0.001). Following a 24 h exposure to N/Se@TCsNPs, planktonic cell counts rose from undetectable levels at OD_595_nm to over 0.2, and this count further increased within 24 h to over 0.6 (*P* < 0.003) (Fig. 5K). Within a 24 h biofilm study, our findings indicate that planktonic cells detached from biofilms are more sensitive to N/Se@TCsNPs treatments compared to those remaining in the biofilm matrix. This observation reinforces the idea that the biofilm life cycle offers protection against antimicrobial substances, while free-floating planktonic cells are more susceptible to such treatments. This vulnerability of planktonic cells further highlights the potent bactericidal action of the developed nanostructure.

## Discussion

In the face of the growing menace of antibiotic resistance, there is an urgent need to evaluate and implement innovative approaches to combat bacterial pathogens [39].

Nisin is produced by *Lactococcus lactis* and has attracted considerable interest due to its low likelihood of promoting the development of bacterial resistance, and its low cellular cytotoxicity at antimicrobial concentrations [40]. This polycyclic peptide antibiotic is more effective against gram-positive bacteria due to differences in cell wall compositions and structures, resulting in lower potency against gram-negative bacteria [41]. The development of bacterial resistance poses a challenge to the application of nisin as an antimicrobial agent. Additionally, the effectiveness of nisin is pH-dependent, with optimal activity observed at acidic pH levels (˂6.0). This limitation restricts its use as an oral drug, as it may not effectively target enteric pathogens [42]. In this study we use nisin in company with selenium to improve antimicrobial and antibiofilm properties of it. The reason for choosing selenium to be combined with nisin is its countless unique properties. First and most aspect that attract our attention in this study is small size of SeNPs allows them to interact more closely with the surface of bacterial cells, which can lead to cellular damage and eventual cell death [43]. Application of TCs as carrier is our second hypothesis that can concentrate nisin at the site of infection and improve its efficacy and reduce the risk of resistance to develop composite. As the previous studies, Cs has shown poor adherence to the mucus layer of the gastrointestinal tract due it precipitation at pH levels above 6.5 in the jejunum, ileum, and colon, which results in inadequate drug absorption [44]. Interestingly, our in vitro result, in accordance with other studies, demonstrated that the modification of Cs, such as TCs, improves mucoadhesive and drug release in the pH of the intestine [45]. Our study revealed a notable difference in the release rate of the nanoparticle formulation between pH = 7.5 and acidic pH conditions. Specifically, the release of the active components from the NPs was observed to be more pronounced in alkaline pH environments than in acidic pH environments. This finding is of significant importance because it highlights the pH-dependent behavior of the formulated composite and its potential for targeted drug delivery. The inclusion of disulfide linkages in the N/Se@TCsNPs structure is instrumental in safeguarding the integrity of the active components, including nisin and selenium, against degradation at lower pH levels [46]. Unlike traditional antibiotics, which primarily target DNA replication or the synthesis of bacterial cells, nisin functions by binding to specific cell wall proteins, altering the membrane’s integrity, and disrupting bacterial metabolism, which ultimately leads to cell death [47, 48]. Nisin also creates pores across the membrane that deplete the proton-motive force, release cytoplasmic components, and alter various energy-dependent cellular processes, all contributing to bacterial death [49].

Studies have demonstrated the inhibitory effects of selenium on the growth of various pathogenic bacteria, such as *S. aureus, E. coli*, and *Helicobacter pylori*. Furthermore, selenium has the ability to augment the antibacterial properties of conventional antibiotics, providing a dual mode of action that may be particularly effective against drug-resistant bacteria [15, 50]. Moreover, the antimicrobial potential of SeNPs extends beyond their direct bactericidal activity. SeNPs have been recognized for their capability to impede the dissemination of environmental antibiotic resistance genes. This advantageous feature offers effective antibacterial properties without adding complexity to the scaling-up and harvesting processes. Consequently, the utilization of SeNPs presents a strategic approach in the battle against antibiotic resistance, as it not only eliminates bacteria but also hinders the propagation of resistance mechanisms [51]. Incorporation of selenium in N/Se@TCsNPs composite, improved bactericidal effects of nisin in a way of inducing oxidative stress in bacteria and pore formation. The effectiveness of SeNPs against gram-negative bacteria stems from their distinct antimicrobial mechanisms, including pore formation, generation of reactive oxygen species, and targeting capabilities. These properties enable SeNPs to overcome the limitations of nisin, such as rapid degradation and limited penetration, thereby increasing their efficacy against various bacteria, including gram-negative strains [52, 53]. Our results confirmed antimicrobial activity of N/Se@TCsNPs can be attributed to the synergism of nisin and selenium as well as controlled release of selenium and nisin facilitated by TCs. N/Se@TCsNPs demonstrated exceptional efficacy against challenging enteric pathogens including *V. cholerae* O1, *C. jejuni*, *S. enterica* subsp. *enterica*, and *S. dysenteriae* (MIC values are 12.5 ± 1.01, 25 ± 4.04, 12.5 ± 0.26, *and* 6.25 ± 0.05 mg/mL, respectively*).* Under these conditions, we consider selenium damages bacterial cell walls and depletes glutathione. This synergistic action of nisin and selenium facilitates the penetration and binding of nisin to the bacterial cell wall, leading to a faster collapse of the wall and subsequent bacterial eradication. Different studies have successfully investigated the synergistic antimicrobial effects of nisin and other components and antibiotics, such as oxacillin [4] (MIC value 4–16 µg/mL), buforin І [54] (MIC value 4–512 µg/mL), Zein [55] (MIC value 7.06–28.25 µg/mL), and garvicin KS, farnesol and polymyxin B [56] (FIC = 0.33), against gram-positive pathogens. The effectiveness of nisin and nisin-loaded NPs varies primarily due to their delivery systems and efficacy in combating bacterial infections. When nisin is encapsulated in nanoscale formats, such as extra-small nisin NPs, it demonstrates enhanced antibacterial activity compared to free nisin. A study conducted by Haider et al. revealed that nisin NPs remain stable at a pH of 5.0 and exhibit higher antimicrobial activity than free nisin at concentrations below 2.0 mg/mL even after autoclave treatment. Additionally, the encapsulation of nisin in solid lipid NPs has been proven to significantly enhance its antimicrobial, antibiofilm, and anticancer effects [57].

According to our findings, the use of TCs can enhance the effectiveness of antibiotics in combating biofilms. TCs facilitate the penetration of antibiotics into biofilms, which is especially advantageous when dealing with bacteria that have developed resistance to traditional antibiotics due to the presence of a protective extracellular matrix within biofilms. The positively charged characteristic of CsNPs enhances their affinity to bind with the negatively charged cell membranes of pathogenic bacteria. This cationic property enables the CsNPs to shield themselves from degradation by glycoside hydrolase enzymes, such as chitinases or chitosanases, that are produced by bacteria. This protective mechanism ensures the integrity and efficacy of the NPs in specifically targeting and disrupting biofilms [58].

**Fig. 6:**
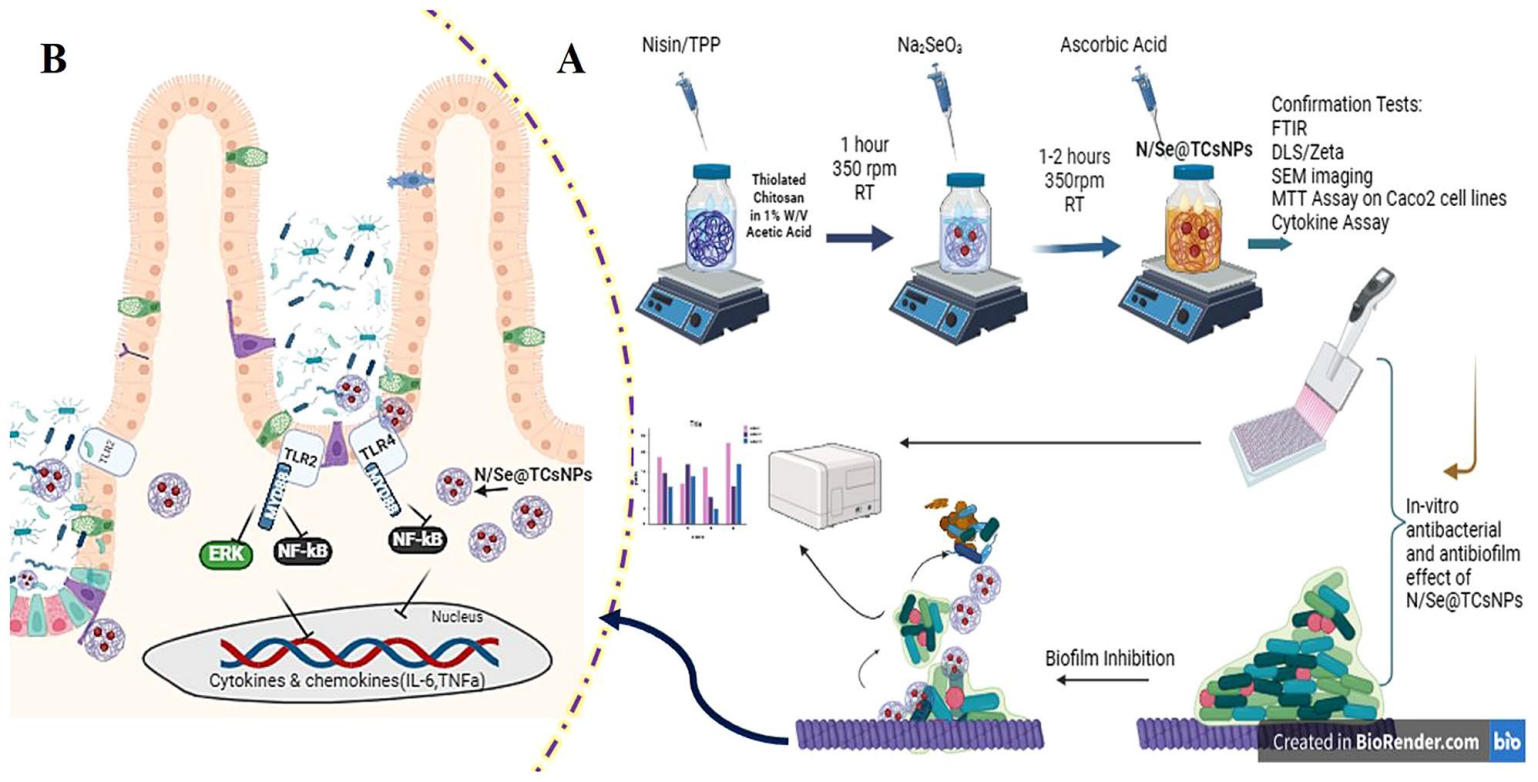
Overview on N/Se@TCsNPs evaluation and in vitro cellular effects. **A**) Results indicated antibacterial/ antibiofilm/anti-attachment effects of designed N/Se@TCsNPs on common enteric pathogens including; *V.cholerae* O1 *El Tor* ATCC 14,035, *C. jejuni* ATCC 29,428, *S.enterica* subsp. *enterica* ATCC 19,430, *S.dysenteriae* PTCC 1188, *E.coli* O157:H7 ATCC 25,922, *L.monocytogenes* ATCC 19,115, and *S.aureus* ATCC 29,733. **B** N/Se@TCsNPs show immunomodulation effects on *TLR2/4*. **N/Se@TCsNPs**: Nisin and selenium encapsulated in chitosan nanoparticles, **TLR**: Toll-like receptor

The ability of N/Se@TCsNPs to reduce bacterial attachment is of great significance (p˂0.0001). Bacterial attachment is a crucial step in the formation of biofilms, which are complex communities of bacteria embedded in a protective matrix. Our results revealed that more than 60% of the tested enteric pathogen isolates are effectively inhibited at a concentration of 3.12 ± 0.17 mg/mL of N/Se@TCsNPs. This dose exhibits a significant biofilm inhibitory effect, equivalent to 1/4 MIC, for *S. enterica* subsp. *enterica* and *V. cholerae* O1 El Tor. Additionally, it demonstrates inhibitory effects at 1/8MIC and 1/2MIC values for *C. jejuni* and *S. dysenteriae*, respectively (*p* < 0.0001). These results underscore the potent antimicrobial activity of N/Se@TCsNPs against the tested enteric pathogens, particularly in inhibiting biofilm formation (Fig. 6A).

In a study in 2023 [59], which is in line with our study, it was shown that the synergism of nisin and p-coumaric acid inhibited the formation of biofilms and reduced the expression of the *SprE* virulence gene in *Enterococcus faecalis* bacteria. Sharafi et al. [60]. reported antibiofilm effect of nisin in combination with oxacillin against *staphylococcus epidermidis*. In another investigation, nisin loaded in CsNPs functionalized with DNase I has shown significant antibiofilm effect on *L.monocytogenes* on food contact surfaces (3 log colony-forming unit (CFU)/cm^2^ reduction) [61]. Several other attempts revealed synergistic antibiofilm properties of nisin in combination with other components [62–66].

Nisin has been shown to modulate the immune response of host by influencing cytokine production. it can increase interleukin IL-10 production and decrease pro-inflammatory cytokine levels, leading to immunomodulatory effects [67]. In human HaCaT keratinocytes stimulated by nisin at a concentration of 25 mg/mL, Mouritzen et al. [68]detected reduced production of pro-inflammatory cytokines (IL-6, IL8, and TNF-α). Nisin increased the anti-inflammatory cytokine (IL-10) and diminished the secretion of pro-inflammatory cytokines (TNF-α, IL-1, and IL-6) in LPS-induced MCF10A cells. Nisin Z also hindered the induction of the ERK1/2 and p38 mitogen-activated protein kinase signaling processes [69]. SeNPs have been also found to have anti-inflammatory effects by inhibiting the nuclear translocation of the NF-B pathway, which controls the production of cytokines, including IL-6 and TNF [70]. In a study conducted by Alkhudhayri et al., it was discovered that SeNPs have the potential to effectively alleviate inflammation in jejunum due to bacterial infections [71]. The results of current study revealed anti-inflammatory nature of formulated N/Se@TCsNPs. In vitro analysis showed when Caco-2 cells were stimulated by N/Se@TCsNPs, the production of TNF-α and IL-6 was markedly decreased (p˂ 0.05), but TGF-β and IL-10 production increased (p˂ 0.001) in comparison to N@TCsNPs and Se@TCsNPs. These findings indicated that the combination of nisin and selenium exhibits a potent synergistic anti-inflammatory effect. Considering the importance role of *TLR2* and *TLR4* in immune response and inflammation, our results indicated significant down regulation of *TLR2* (0.25 ± 0.09-fold) and *TLR4* (0.38 ± 0.03-fold) gene expression in response to N/Se@TCsNPs treatment which supported anti-inflammatory properties of developed nanostructure (Fig. 6B).

## Conclusion

In conclusion, the results obtained from this investigation reveal the strong antibacterial and antibiofilm properties of N/Se@TCsNPs against challenging enteric pathogens, including *V. cholerae* O1 El Tor, *C. jejuni, S. enterica* subsp. *enterica, S. dysenteriae, E. coli* O157:H7, *L. monocytogenes*, and *S. aureus*.

The remarkable effectiveness of the developed nanomedicine, N/Se@TCsNPs, is evident as it exhibits potent bactericidal effects without the need for antibiotics. This underscores the superiority of our designed nanomedicine in combating infections caused by both gram-negative and gram-positive bacteria. The use of TCs as carriers has not only enhanced the bio-distribution and pharmacokinetics of nisin and selenium but also increased their resistance to degradation. Further research is anticipated to provide valuable insights into the various aspects of this novel nanomedicine in in vivo models, particularly as an oral drug.

## Data Availability

The datasets used and/or analyzed during the current work are available upon reasonable request from the corresponding author.

## Abbreviations

N/Se@TCsNPs: Nisin and selenium encapsulated in thiolated chitosan nanoparticles
N@TCsNPs: Nisin encapsulated in thiolated chitosan nanoparticles
Se@TCsNPs: Selenium encapsulated in thiolated chitosan nanoparticles
SeNPs: Selenium nanoparticles
Caco-2: Human colorectal adenocarcinoma cells
MTT assay: (3-(4,5-dimethylthiazol-2-yl)-2,5-diphenyltetrazolium bromide)
Cs: Chitosan
CsNPs: Chitosan nanoparticles
TCs: Thiolated chitosan
FTIR: Fourier-transform infrared spectroscopy
DLS/Zeta: Dynamic light scattering/Zeta potential
SEM: Scanning electron microscopy
TGA/DSC: Thermal gravity analysis/ Differential scanning calorimetry
TLR: Toll-like receptor
qRT-PCR: Quantitative real-time PCR
